# Anesthetics and epigenetics

**DOI:** 10.18632/oncotarget.13143

**Published:** 2016-11-05

**Authors:** Nilkantha Sen

**Affiliations:** Department of Neuroscience and Regenerative Medicine, Augusta University, Augusta, GA, USA

**Keywords:** CREB, HDAC4, anesthetic

Developmental brain in humans and rodents is complex in nature and is composed of continuous alteration of neuronal proliferation, synaptogenesis, synaptic plasticity density to establish and maintain the complicated networks in the brain and central nervous system. The developing brain always being exposed to several environmental challenges including anesthetics that can significantly affect the existing network in the brain and force them to reorganize the network to accommodate the changes. It was reported that millions of infants and children are exposed to anesthetics each year all over the world. Moreover, more than 2% pregnant women are exposed to anesthetics for the issues which are not related to delivery. The most unfortunate scenario is that exposure to anesthetics to developing brain induces several deleterious effects at the molecular and cellular levels which include neuronal apoptosis that could affect the developmental network in the brain and leads to cognitive dysfunctions and behavioral changes [1–3]. Most importantly these effects can be harmful and long-lasting in their life. A retrospective cohort study investigated more than 5000 children and found that children who had undergone anesthesia and surgery before 3 years of age were nearly twice as likely to be diagnosed with developmental and behavioral disorders compared with children who had never undergone anesthesia [4]. Consistent with this study it was found that exposure to anesthesia such as isoflurane causes an increased risk for learning and memory disability in the developing brain [5] and serve as an independent risk factor for neurobehavioral impairment due to memory disability.

In general, memory is defined at the cellular level by activity-dependent modulation of both the structure and the function of specific synaptic connections that in turn, depends on the activation of a specific pattern of gene expression regulated by the status of chromatin and activation of transcriptional factors. Chromatin remodeling, especially through histone tail acetylation, which alters the compact chromatin structure and changes the accessibility of DNA to transcription factors, is emerging as a fundamental mechanism for regulating gene expression related to memory. Recently we have shown that administration of anesthetics such as isoflurane leads to downregulation of genes responsible for memory and cognitive functions, such as BDNF, and c-fos in the developing brain of mice due to transcriptional inactivation of a transcription factor, CREB [6]. The CREB family of transcription factors are involved in controlling the transcriptional responses to a wide range of extracellular signals in neurons. In particular, CREB has been involved in many aspects of nervous system function, from activity-dependent synaptic plasticity during development and in the adult brain, to neuronal survival. CREB participates in numerous cell processes and stands at the crossroad of different signaling pathways. Synaptic activity, hormones, growth factors released during development, hypoxia and stress, among other stimuli, can trigger the phosphorylation of CREB, causing its activation and subsequent induction of CREB-dependent gene expression. The phosphorylated form of CREB (pCREB) then recruits the coactivator CREB-binding protein (CBP), which triggers transcription initiation by both bringing the RNA polymerase II complex to the promoter and acetylating histones in chromatin [7].

To elucidate the molecular mechanism how exposure to anesthetics causes impairment of CREB and neurite outgrowth, we found that exposure to isoflurane leads to an increase in nuclear translocation of a histone deacetylase 4,HDAC4, which interacts with CREB in the nucleus. This event, in turn, results in a decrease in interaction between an acetyltransferase, CBP, and CREB that ultimately leads to transcriptional inactivation of CREB. As a result, the expression level of BDNF, and c-fos were significantly down-regulated after administration of isoflurane in the developing brain [6]. The HDACs are one of the major regulators of histone acetylation and synaptic plasticity. Within the Class, I HDACs, HDAC 1, 2, and 8 are found primarily in the nucleus, whereas HDAC3 is found in both the nucleus and the cytoplasm and is also membrane-associated. Class II HDACs (HDAC4, 5, 6, 7 9, and 10) are able to shuttle in and out of the nucleus, depending on the signals. However, only HDAC4, 5 and 9 are present in the brain [8]. Our results provide a novel mechanism where HDAC4 impairs transcriptional activation of CREB without altering the acetylation status of histones [6]. Thus, our study provides a mechanistic framework on which HDAC4 inhibitor can be tested as a therapeutic approach to prevent neurotoxicity upon exposure to anesthetics.
